# Measuring hospital performance in the management of acute coronary syndromes

**DOI:** 10.1007/s12471-014-0570-8

**Published:** 2014-06-25

**Authors:** R. J. de Winter

**Affiliations:** Department of Cardiology, B2-137, Academic Medical Center, Meibergdreef 9, 1105AZ Amsterdam, the Netherlands

In recent years it has become customary to publish the design of a clinical trial at the time that the trial commences. When the results of the trials become available for publication at a later time point, the methods can be described briefly, while referring to the previously published design paper. In this issue of the journal, Tra et al. [1] publish the design of a retrospective study (not a clinical trial) evaluating adherence to guideline-recommended management in patients with acute coronary syndromes (ACS) in 13 Dutch hospitals in 2012.

An expert panel identified three recommendations: timely invasive management in ST-segment elevation myocardial infarction (STEMI) patients, use of a validated risk score in non-STEMI/unstable angina (NSTEMI/UA) patients and prescription of secondary prevention medication (the golden five) at discharge in ACS patients. Hospital chart review, abstracting of patient records and data from hospital information systems will be collected by trained data abstracters. The primary endpoint will be the percentage of patients achieving reperfusion within 90 min from first medical contact, percentage of patients in whom a validated risk score was used and percentage of patients discharged on acetylsalicylic acid, thienopyridine, statin, beta blocker, and ACE inhibitor. Importantly, contraindications for prescription of medication will be recorded.

The use of a retrospective, cross-sectional design to evaluate the quality and performance of hospitals in the management of patients with ACS has several important shortcomings. The retrospective nature of the data collection will result in many missing items. The information in the patients charts is recorded for clinical purposes, not for performance measures, and will therefore be subject to all kinds of bias and confounding. ACS patients are a particular heterogeneous population that may have a very dynamic pre-hospital, in-hospital and post-discharge clinical course which is difficult to reconstruct retrospectively. This is difficult for cardiologists with years of clinical experience, as evident from discussions in clinical endpoint committees in ACS trials, and this may even be more difficult for data abstracters trained in social sciences. Prospective collection of performance measures has been performed previously in large studies, but this provides only a distant reflection of quality of patient care. It is extremely difficult to measure quality of care even in a prospective design, so one is left with what we can measure, such as time between first medical contact to balloon within 90 min yes/no, use of risk score yes/no or prescription of discharge medication yes/no. The retrospective design of the present study represents a serious drawback, but also highlights a sobering fact: for years it has been impossible to organise a proper, prospective, nationwide, validated ACS registry in the Netherlands that includes the three proposed study endpoints. It is likely that in the Netherlands, most patients with STEMI will be recognised in the ambulance with high-quality field-ECG and within a regional STEMI network these patients will undergo primary percutaneous coronary intervention (PCI) as soon as possible. PCI centres generally record their own performance including time intervals, but we do not have any national data. I am sure that most ACS patients are evaluated using validated risk scores to drive clinical decision-making and discharge medication will follow the recommended guidelines, but we do not know for sure. The most striking aspect of the study design by Tra et al. is the fact that it exposes a major embarrassment, we do not have a national ACS registry, whereas this has been possible for many years in other countries such as Sweden and the UK [2].

For each of the three ‘endpoints’, one can argue that these only remotely reflect quality of care. Timely reperfusion in patients with STEMI is important and related to prognosis, but whether the 90 min time interval is useful is debated. The time stamp for first medical contact may be the time of arrival of the ambulance, not the time of diagnosis. Time of first ECG is usually a reliable time stamp, but may not be available in many patients in the patient records. Depending on regional travel distances in more rural areas, first medical contact-to-needle times represents geography instead of quality of care. In a recent study by Menees et al. [3] in the New England Journal of Medicine comprising 96,738 admissions for primary PCI in STEMI patients, door-to-balloon times were reduced from 83 to 67 min without any change in in-hospital mortality. One can argue that *avoidable delays* within a STEMI network may be a better performance measure. Or even more important, the way care is delivered to complex and very sick patients, such as patients in cardiogenic shock, patients with recurrent ventricular arrhythmias in the setting of STEMI, the recognition of patients with STEMI equivalents without overt ST elevation on the ECG or patients with aortic dissection presenting with STEMI-like symptoms.

The use of a validated scoring system in patients with NSTEMI/UA was implemented in most hospitals after the recommendation was included in the European Society of Cardiology nSTE-ACS guidelines [4]. The GRACE score has been validated in large cohorts and holds prognostic information that can guide the more invasive approach in high-risk patients [5]. In particular, a GRACE score > 140 is currently used to select patients to undergo angiography within 24 h after the diagnosis, although some argue that there is at present insufficient prospective evidence to support this 24-h time window [6]. Using some form of risk assessment in an individual patient, prior to choosing an early invasive or more selective approach, including estimating the potential bleeding risk associated with invasive procedures, is what cardiologists do. Documenting all the components of complex medical decision-making is important, but does not replace it. The burden of registering data seems to increase incessantly. The amount of time clinicians are already spending behind computer screens instead of at the bedside with their patients is out of proportion and in itself is becoming a threat to quality of care.

Finally, discharge medication for secondary prevention for ACS patients should include dual antiplatelet therapy and statins. The prescription of beta blockers and ACE inhibitors can be individualised, depending on left ventricular function, renal function, age, the presence of diabetes and the ECG (heart frequency at rest and AV-conduction characteristics). Beta blockers or ACE inhibitors have a class I level of evidence A recommendation for patients with impaired left ventricular function with or without symptoms. [4] It will be very interesting to see whether contraindications for the use of beta blockers and/or ACE inhibitors can be recovered from the patient charts.

In summary, the study by Tra et al. is a courageous attempt to collect data retrospectively that we should have available in a prospective database. Such a prospective registry is costly and difficult in an already overstretched system. The challenge for Dutch cardiologists will be to implement a national ACS registry with reliable data in an environment where already so much time is spent with data collection, registration, and documentation for all sorts of purposes that eats away at time available for the delivery of patient care.
